# Facilitating Maize Seed Germination Under Heat Stress via Exogenous Melatonin

**DOI:** 10.3390/ijms26041608

**Published:** 2025-02-13

**Authors:** Congcong Chen, Dongxiao Li, Yujie Yan, Congpei Yin, Zhaojin Shi, Yuechen Zhang, Peijun Tao

**Affiliations:** College of Agriculture, Hebei Agricultural University, Baoding 071001, China; 15733227268@163.com (C.C.); lidongxiao.xiao@163.com (D.L.); yyj1853267@163.com (Y.Y.); ycongpei2015@163.com (C.Y.); shizhaojin1998@163.com (Z.S.)

**Keywords:** maize, seed germination, melatonin, heat stress, molecular mechanisms

## Abstract

Seed germination is a critical phase during which plants are particularly sensitive to environmental stresses, especially heat stress, due to the high metabolic and physiological activities required for initial growth. Melatonin (MT), a key antioxidant, is crucial for assisting plants in managing abiotic stresses. While the impact of melatonin on heat stress has been explored in other developmental stages or species, this is the first study to specifically focus on its role during maize seed germination under heat stress. The treatment with 50 μM melatonin significantly enhanced seed germination under heat stress by improving antioxidant capacity, osmotic regulation, and hydrolytic enzyme activity, likely through the modulation of key signaling pathways, thus reducing oxidative damage and starch content. Furthermore, melatonin application promoted the accumulation of endogenous gibberellins (GAs) and significantly inhibited abscisic acid (ABA) content, thereby maintaining a dynamic equilibrium between these phytohormones. Principal component analysis and correlation analysis provided deeper insights into the overall effects of these physiological and biochemical parameters. Integrated transcriptomic and metabolomic analysis revealed that melatonin exerted its regulatory effects by modulating key genes and pathways associated with antioxidant defense, stress responses, and plant hormone signal transduction. Furthermore, melatonin significantly modulated the GA and ABA signaling pathways, starch and sucrose metabolism, and phenylpropanoid biosynthesis, thereby reducing oxidative damage induced by heat stress and strengthening the defense mechanisms of maize seeds. The alignment between the qRT-PCR findings and transcriptomic data further validated the robustness of these underlying mechanisms. In conclusion, this study provides novel insights into the role of melatonin in enhancing maize seed germination under heat stress and offers a promising strategy for improving crop heat tolerance through melatonin application in agricultural practices.

## 1. Introduction

Heat stress (HS) is a significant abiotic stress that plants face, and due to the intensification of global climate change, it has emerged as a crucial limiting factor for crop growth and agricultural yield [1]. In response to heat stress, plants have evolved a range of intricate mechanisms to enhance their heat tolerance [2]. Heat stress greatly increases the concentration of reactive oxygen species (ROS) within plant cells. An overaccumulation of reactive oxygen species, such as the superoxide anion (O_2_^−^) and hydrogen peroxide (H_2_O_2_), can lead to oxidative harm, endangering vital biomolecules like cell membranes, proteins, lipids, and DNA [3,4]. This overproduction of ROS is typically caused by dysfunctions in the mitochondrial respiratory chain and plasma membrane. In mitochondria, elevated temperatures reduce ATP synthesis, severely disrupting cellular energy metabolism [5]. In order to reduce oxidative harm, plants initiate several antioxidant defense mechanisms, which comprise enzymes like superoxide dismutase (SOD), catalase (CAT), and peroxidase (POD), along with compounds like ascorbic acid (AsA) and glutathione peptides. Additionally, non-enzymatic antioxidants, including glycopeptides (GSH), serve to neutralize reactive oxygen species (ROS) [6,7,8,9]. Moreover, heat stress also impacts the structure of plant cell walls and their osmotic regulation capacity. Alterations in the synthesis of osmotic regulators, such as soluble sugars and proline, affect the water balance, thereby exacerbating cellular damage under heat stress [10]. Heat stress can also induce the misfolding of proteins within cells, which not only disrupts their normal function but also leads to the activation of heat shock proteins (HSPs) and proteasomal pathways, depleting cellular resources and triggering programmed cell death (PCD) [11,12]. In addition, heat stress modulates gene expression regulation. For instance, in *Arabidopsis*, heat stress induces the expression of heat shock factor *HSFA1s*, thereby enhancing its heat tolerance [13]. In rice, three major heat tolerance QTLs (quantitative trait loci) have been identified, namely THERMO-TOLERANCE1 (*TT1*), *TT2*, and *TT3*, which can significantly increase grain yield under heat stress conditions [14,15,16]. *TT2* can also work with MYB5 to modulate the *HSFA2* promoter in heat stress, enhancing heat stress tolerance in plants, and SIIE elements play a key role in this process [17]. Furthermore, heat stress activates *MAPK6*, enhancing its activity, which in turn regulates PCD in plants. Subsequent studies have revealed that not only *MAPK3/6* but also *MAPK4* can phosphorylate *HsfA4a*, regulating its molecular interactions. The overexpression of *HsfA4a* significantly improves heat tolerance in plants by minimizing oxidative damage [18].

Melatonin (N-acetyl-5-methoxytryptamine) is an indole compound that demonstrates regulatory functions akin to those of plant hormones [19,20] and plays a crucial role in several phases of plant growth and development, such as seed germination [21,22], flowering [23], chlorophyll production [24], and the postponement of leaf senescence [25]. In addition to its regulatory functions, melatonin is widely recognized for its potent antioxidant properties, bolstering plant resilience to abiotic stresses and mitigating oxidative damage caused by environmental stress. Research has demonstrated that 100 μM melatonin enhances heat tolerance in rice seeds by stimulating antioxidant enzyme activity. In contrast, 70 μM melatonin notably enhances maize seedling survival under heat stress [26]. Furthermore, studies on other crops reveal that melatonin reduces malondialdehyde (MDA) content, enhances photosynthetic efficiency, and preserves cell membrane integrity, thereby enabling plants to sustain physiological functions under extreme temperature, salt, drought, and UV stress conditions [27,28,29,30,31]. These protective effects are primarily attributed to its ability to modulate endogenous hormone levels, activate antioxidant enzymes, and inhibit the accumulation of ROS, establishing it as a promising exogenous regulator in the study of stress resilience. Although the role of melatonin in mitigating heat stress has been explored in various crops, its specific effects during maize seed germination, particularly under high-temperature conditions, remain poorly understood. This study systematically investigated the changes in transcription and metabolism during maize seed germination under heat stress, exploring the underlying mechanisms of melatonin’s protective effects.

Maize, one of China’s three main staple crops, is crucial for national food security. Consistent yield growth is essential for meeting the country’s increasing demand [26]. Summer maize is particularly popular across various provinces because of its short growth cycle and its ability to complement post-harvest autumn crops, thereby enhancing land use efficiency [32]. Seed germination is the most susceptible stage in the plant growth cycle, rendering plants particularly vulnerable to environmental stresses [26]. Unfavorable conditions during this stage can significantly reduce germination rates, impair seedling quality, and ultimately lower crop yields [33]. Summer maize is especially prone to heat stress, as it is grown in a period of high temperature from early to mid-June. This period aligns with key growth stages, such as seed germination and early seedling development, when plants are highly vulnerable to environmental stressors [34]. Heat stress exacerbates oxidative stress, accelerating metabolic rates and disrupting the hormonal balance, which can delay or inhibit germination [35,36]. Thus, implementing strategies to mitigate heat stress during seed germination is critical for ensuring uniform maize emergence and maximizing its yield potential [3].

This research intends to explore the mechanisms through which external melatonin promotes the germination of maize seeds when subjected to high temperatures. The emphasis is on investigating how melatonin influences the antioxidant capacity, osmotic balance, activity of hydrolytic enzymes, and balance between the GA and ABA hormones. Furthermore, transcriptomic and metabolomic analyses are employed to identify crucial genes and pathways that play a role in stress responses and defense mechanisms. The results provide fresh perspectives on the potential use of melatonin as a means of enhancing heat tolerance in crops.

## 2. Results

### 2.1. Exogenous Melatonin Promotes Maize Seed Germination

**Figure 1:**
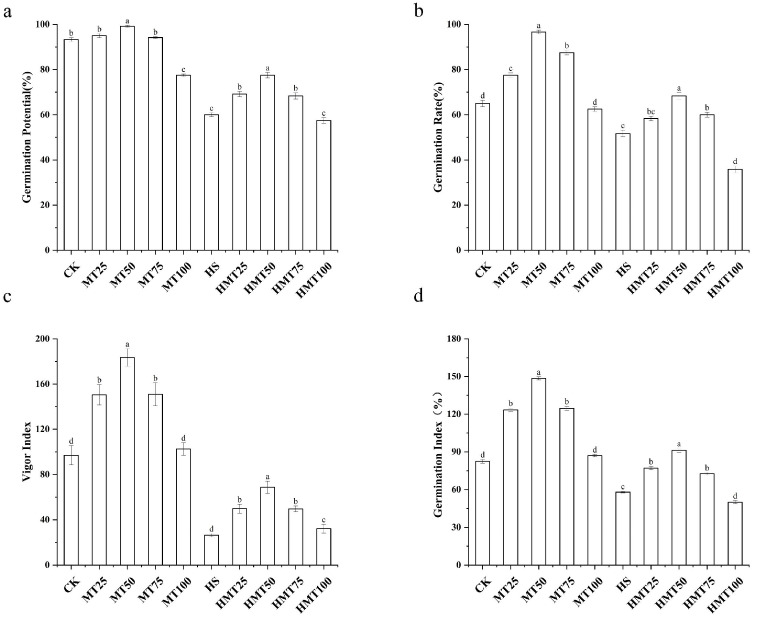
Effects of melatonin on maize seed germination under HS. (**a**) Germination rate. (**b**) Germination vigor. (**c**) Germination index. (**d**) Seed vitality. The letters on the bar chart represent different levels of significance, with *p* < 0.05.

A series of experimental analyses were conducted to evaluate the effects of exogenous melatonin on maize seed germination across varying concentrations (Figure 1). It was found that, under normal temperature conditions, seed germination rates were consistently high for all melatonin treatments, with the exception of the 100 μM concentration, which exhibited a significantly lower germination rate. These results indicate that melatonin at moderate concentrations could promote seed germination. In contrast, heat stress significantly inhibited maize seed germination, resulting in a 33.33% decrease in the germination rate. Remarkably, the HMT-50 treatment resulted in a 17.5% increase in the germination rate compared to the HS treatment alone. The germination potential, germination index, and seed vigor followed a similar pattern. These results suggest that an optimal concentration of melatonin not only enhances seed germination under normal conditions but also offers protective effects under stress conditions. Based on these findings, a melatonin concentration of 50 μM was selected for further investigations.

### 2.2. Exogenous Melatonin Promotes the Growth of Maize Radicles and Plumules

**Figure 2:**
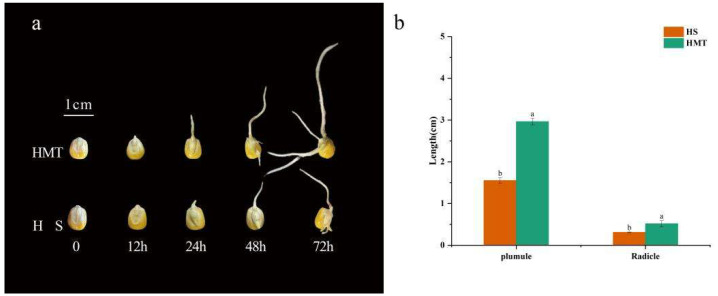
Effects of melatonin on maize seed morphology under HT. (**a**) Phenotypic analysis of seeds after 12, 24, 48, and 72 h under HS and HMT treatments. (**b**) Plumule length and radicle length. The letters on the bar chart represent different levels of significance, with *p* < 0.05.

Phenotypic analysis was performed on maize seeds exposed to heat stress for 12, 24, 48 and 72 h (Figure 2a). The results revealed that after 12 h of HS treatment, the HMT treatment seeds germinated earlier than the HS treatment seeds, while the seeds under HS showed no signs of germination. By 24 h, although the seeds under the HS treatment had begun to germinate, their radicle development was significantly delayed, exhibiting stunted and poorly developed roots. In contrast, the HMT treatment showed a notably faster germination rate and improved resistance to stress. After 72 h, the lengths of the radicles and plumules were measured with a caliper. The results indicated that heat stress significantly inhibited the growth of both the radicles and plumules. However, the radicle length in the HMT-treated seeds was nearly twice that of the HS-treated seeds, while the plumule length increased by 60.78% (Figure 2b). Additionally, the radicles and plumules of the HMT-treated seeds were thicker.

### 2.3. Exogenous Melatonin Alleviates Oxidative Stress in Maize Seeds

**Figure 3:**
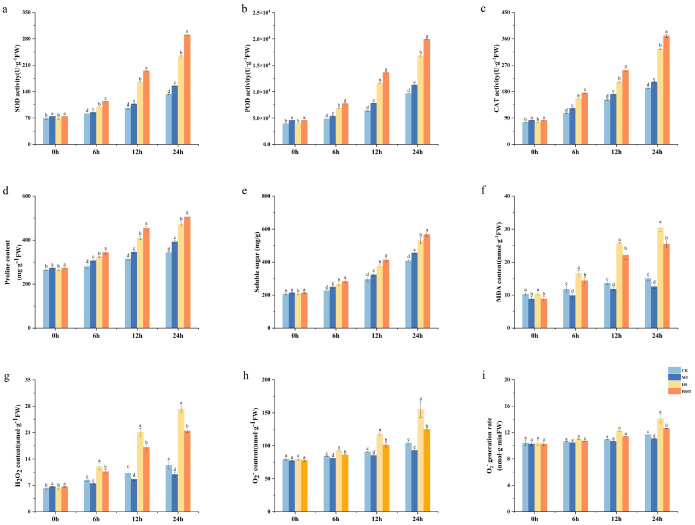
Effects of melatonin treatment on antioxidant enzyme activity, osmotic regulators, and peroxide products under HS. (**a**) SOD activity. (**b**) POD activity. (**c**) CAT activity. (**d**) PRO content. (**e**) Soluble sugar content. (**f**) MDA content. (**g**) H_2_O_2_ content. (**h**) O_2_^−^ content. (**i**) O_2_^−^ rate. The letters on the bar chart represent different levels of significance, with *p* < 0.05.

Figure 3a–c illustrate the changes in antioxidant enzyme activity (SOD, POD, and CAT) during seed germination. Compared to the CK and MT treatments, both the HS and HMT treatments exhibited a sharp increase in antioxidant enzyme activity after 6 h, peaking at 24 h. At this point, the activities of SOD, POD, and CAT in the HS treatment increased by 56.75%, 57.49%, and 59.06%, respectively, compared to CK, indicating that heat stress significantly enhanced antioxidant capacity. Although the increase in the HMT treatment seeds was lower than that in the HS treatment seeds (45.53%, 48.51%, and 51.98%, respectively), the enhancement was still significant, suggesting that melatonin effectively alleviates oxidative damage induced by heat stress.

Additionally, the proline and soluble sugar contents were higher in both the HS and HMT treatments compared to the CK and MT treatments, with peak levels occurring at 24 h. The HS treatment significantly increased the proline and soluble sugar contents by 72.72% and 76.88%, respectively, while the HMT treatment caused increases of 67.92% and 71.91% (Figure 3d,e). These results suggest that melatonin promotes the accumulation of osmotic regulators, which helps to mitigate the negative impact of heat stress on maize seed germination.

Moreover, the levels of MDA, H_2_O_2_, and O_2_^−^ and the O_2_^−^ rate were measured (Figure 3g–i), with peak values observed at 24 h, suggesting that cellular damage was most pronounced at this time. In the HS treatment, MDA, H_2_O_2_, and O_2_^−^ levels increased significantly by 51.2%, 54.66%, and 29.80%, respectively, compared to CK, reflecting significant membrane damage and excessive accumulation of ROS induced by heat stress. In contrast, the increases in the HMT treatment were lower (41.01%, 42.46%, and 16.30%, respectively), indicating that melatonin partially mitigates the excessive generation of ROS.

### 2.4. Melatonin Treatment Increases Galactosidase and Amylase Activity in Maize Seeds

**Figure 4:**
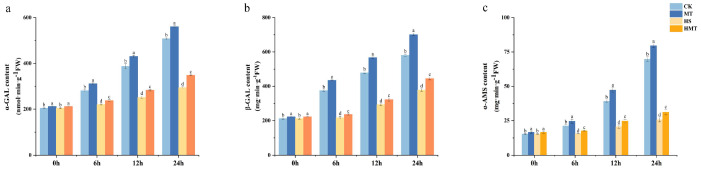
Effects of melatonin on amylase and lactase activity under HS. (**a**) α-GAL activity. (**b**) β-GAL activity. (**c**) α-AMS activity. The letters on the bar chart represent different levels of significance, with *p* < 0.05.

The experimental results demonstrated an overall upward trend in the activities of α-GAL, β-GAL, and α-AMS. Notably, the CK and MT treatments exhibited considerably higher enzyme activities than the HS and HMT treatments. As shown in Figure 4, at 24 h, the enzyme activities in the HS treatment dropped significantly by 63.24%, 42.01%, and 34.85%. In comparison, the HMT treatment exhibited a more moderate decline, with reductions of 55.37%, 31.34%, and 23.29%. Although heat stress suppressed α-AMS activity, the HMT treatment consistently showed higher enzyme activities than the HS treatment. These findings suggest that melatonin partially alleviates the inhibitory effect of heat stress on hydrolytic enzyme activity, thereby promoting starch hydrolysis and carbohydrate metabolism.

### 2.5. Exogenous Melatonin Promotes GA Synthesis and Inhibits ABA Accumulation

**Figure 5:**
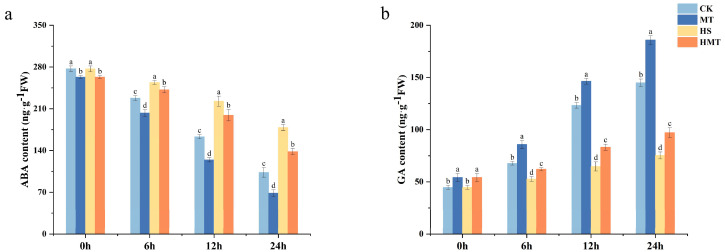
Effects of melatonin treatment on plant hormones in maize seeds under HS. (**a**) ABA content. (**b**) GA content.

The results reveal a downward trend in ABA levels, with the rate of decline varying significantly across treatments (Figure 5a,b). Compared to CK, the MT treatment exhibited the most rapid reduction in ABA content, suggesting that melatonin effectively suppresses ABA accumulation and accelerates seed germination. In contrast, the HS treatment showed the slowest decline, with a 72.90% increase in ABA content at 24 h compared to CK, likely contributing to delayed germination and elevated ABA levels induced by heat stress. However, the increase in ABA content under heat stress with HMT was significantly lower, at only 33.63%, compared to the HS treatment, suggesting that melatonin partially alleviates heat-induced ABA accumulation and mitigates the adverse effects of stress on seed germination. The trend in GA content was opposite to that of ABA, with GA levels progressively increasing during the germination process. Notably, GA levels showed a significant rise at 12 h, reaching a peak at 24 h, suggesting that melatonin may play a positive role in the synthesis or regulation of GA. In contrast, the HS treatment led to a significant reduction (92.48%) in GA levels compared to CK, indicating that heat stress significantly inhibited GA accumulation, thereby suppressing seed germination. However, seeds treated with HMT exhibited a comparatively smaller decrease in GA content, with 49.19%, indicating that melatonin partially restores GA accumulation and mitigates the inhibitory effects of heat stress on GA metabolism.

### 2.6. Comprehensive Analysis of the Effect of Exogenous Melatonin on Maize Seeds

**Figure 6:**
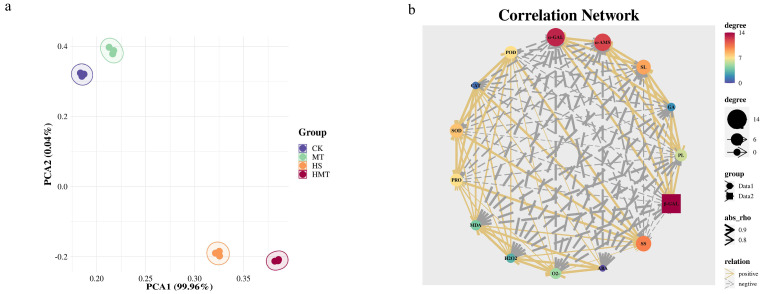
Effects of melatonin on various factors of maize seed germination under HS. (**a**) Principal component analysis. (**b**) Correlation analysis.

PCA revealed distinct physiological and biochemical responses among the different treatment groups. As shown in Figure 6a, PCA1 primarily captured the significant physiological changes induced by heat stress, with the HS group positioned far from the CK and MT groups. This suggests substantial internal physiological alterations in seeds subjected to high temperatures. The HMT group showed partial mitigation along PCA1, suggesting that melatonin partially restores these physiological parameters, though not to the levels observed in CK. Although PCA2 accounted for a minor portion of the variance, the positioning of the HMT group indicates that melatonin induces distinct physiological changes under heat stress. These changes are likely associated with the rebalancing of the antioxidant enzyme system, reduction in lipid peroxidation in membranes, and modulation of hormone levels.

As illustrated in the Figure 6b, Further analysis through a complex correlation network examined the relationships among key indicators and their impact on seed germination, with particular emphasis on changes under melatonin treatment. The observed negative correlation between ABA and GA reflects their antagonistic roles in seed germination. Under heat stress, elevated ABA levels inhibit germination; however, melatonin mitigates this effect by decreasing ABA levels and enhancing GA production, thereby facilitating germination. The negative correlations between MDA, H_2_O_2_, and O_2_^−^ and antioxidant enzymes like SOD, CAT, and POD suggest that melatonin protects seed cellular structure and function under heat stress by reducing the accumulation of ROS. In summary, melatonin enhances seed germination by modulating plant hormone levels, activating the antioxidant defense system, and reducing oxidative damage. This network analysis reveals that seed germination is the result of a synergistic interplay between hormonal regulation and antioxidant activity rather than being driven by a single factor.

### 2.7. Transcriptome Analysis of Differentially Expressed Genes

**Figure 7:**
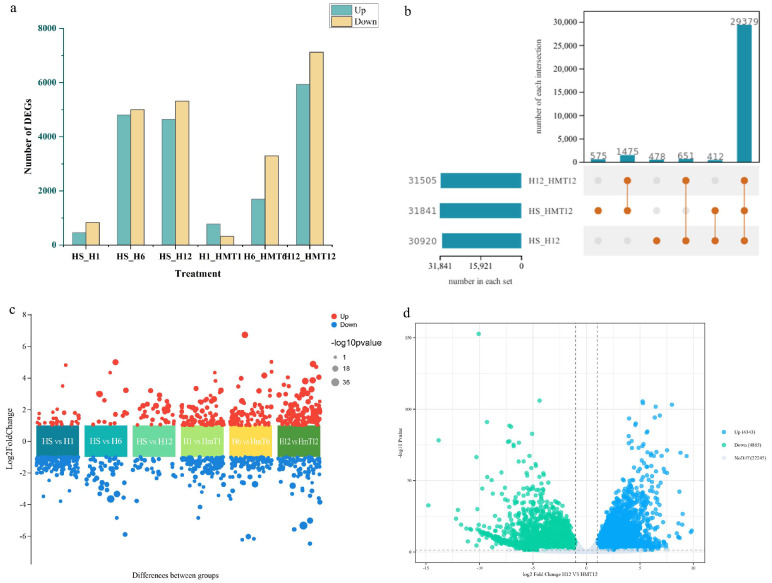
Analysis of differentially expressed genes in maize seeds under HS. (**a**) Bar chart showing the number of DEGs. (**b**) Venn diagram of DEGs. (**c**) Volcano plot illustrating DEGs at 12 h for each group. (**d**) Volcano plot comparing DEGs at 12 h between HS and HMT treatments.

The results reveal notable differences in the number and expression patterns of DEGs between the HS and HMT treatments (Figure 7). At 1 h, 779 genes were upregulated, while 286 genes were downregulated. By 6 h, the number of DEGs had risen significantly, with 1695 genes upregulated and 2881 downregulated. Notably, at 12 h, the number of DEGs increased dramatically, with 5935 genes upregulated and 6232 downregulated, suggesting that melatonin plays a crucial regulatory role during the critical phase of radicle protrusion. These findings suggest that during germination, melatonin initiates germination signals in the early phase (1 h) by regulating a limited set of key genes. In the mid to later stages (6 h and 12 h), it further reinforces and accelerates the process by modulating a vast number of genes, thereby facilitating successful progression through germination.

### 2.8. GO and KEGG Analyses of Differentially Expressed Genes

**Figure 8:**
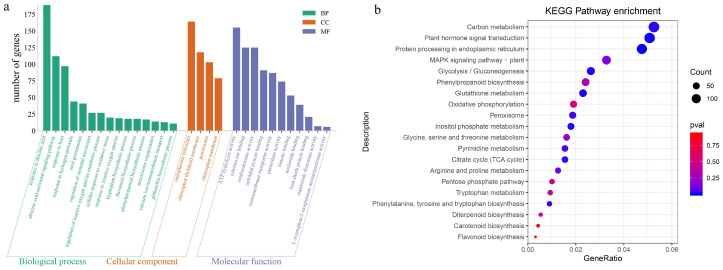
Enrichment analysis of DEGs. (**a**) GO enrichment analysis of DEGs. (**b**) KEGG enrichment analysis of DEGs.

We conducted Gene Ontology (GO) and Kyoto Encyclopedia of Genes and Genomes (KEGG) enrichment analyses of DEGs to explore the key pathways through which melatonin enhances seed germination. The analysis of Figure 8a indicates that in the biological process (BP), significant enrichment was observed in pathways associated with stress response and hormone regulation, including “response to abscisic acid”, “abscisic acid-activated signaling pathway”, “response to heat”, and “response to hydrogen peroxide”. In the cellular component (CC), DEGs were predominantly associated with organelles such as the “endoplasmic reticulum”, “chloroplast thylakoid membrane”, and “peroxisome”. Molecular function (MF) analysis revealed key activities, including “ATPase activity”, “calcium ion binding”, and “oxidoreductase activity”. Enrichment in biological processes was the most pronounced, followed by molecular functions, suggesting that the primary role of melatonin in promoting seed germination involves regulating plant stress responses, antioxidant systems, and hormone signaling. The KEGG enrichment analysis further showed that the DEGs affected by melatonin were significantly enriched in several pathways, including “carbon metabolism”, “plant hormone signal transduction”, “protein processing in the endoplasmic reticulum”, “plant MAPK signaling pathway”, and “glycolysis and gluconeogenesis”, as presented in Figure 8b. These findings emphasize that melatonin enhances the plant’s antioxidant capacity and mitigates stress-induced damage by modulating carbohydrate metabolism, signaling pathways, and the synthesis of secondary metabolites.

### 2.9. qRT-PCR Validation of DEGs

**Figure 9:**
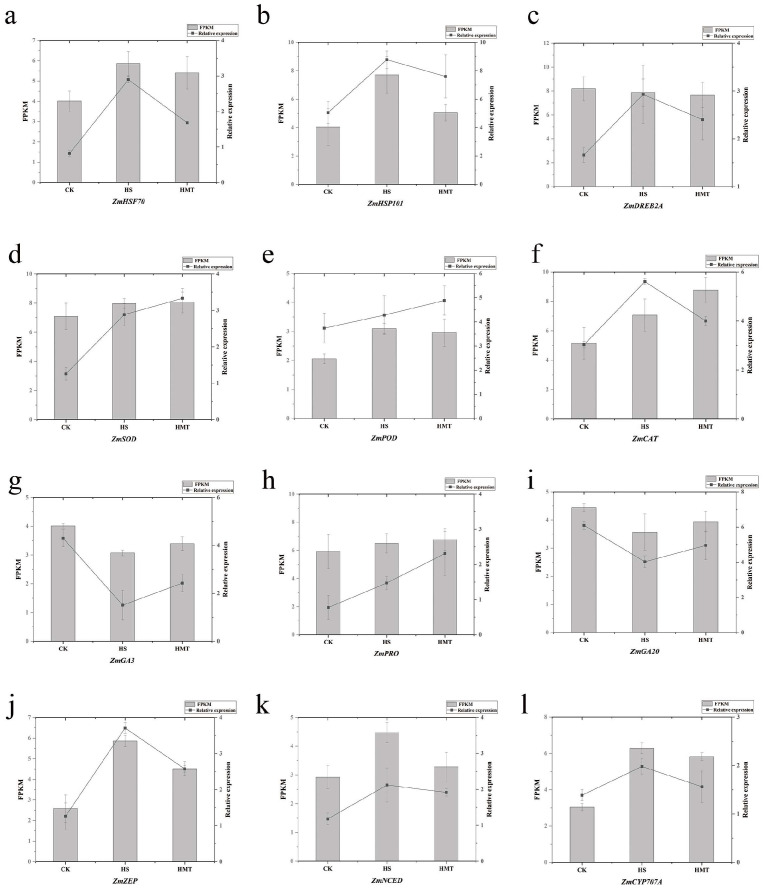
qRT-PCR of 12 DEGs associated with maize seed germination under HS. (**a**) ZmHSF70. (**b**) ZmHSF101. (**c**) ZmDREB2A. (**d**) ZmSOD. (**e**) ZmPOD. (**f**) ZmCAT. (**g**) ZmPRO. (**h**) ZmZmGA3. (**i**) ZmGA20. (**j**) ZmZEP. (**k**) ZmNCED. (**l**) ZmCYP707A.

To validate the accuracy of the RNA-seq data, we selected 12 DEGs, including 3 heat stress-related genes (*HSF70*, *HSF101*, and *DREB2A*), oxidative and antioxidative genes (*SOD*, *POD*, *CAT*, and *PRO*), and hormone metabolism pathway genes (*GA3*, *GA20*, *ZEP*, *NCED*, and *CYP707A*). The expression levels of these genes were verified by qRT-PCR, and the results demonstrated a strong correlation with the transcriptome sequencing data, confirming the reliability of the RNA-seq findings in Figure 9.

### 2.10. Metabolomic Analysis of Maize Seeds

**Figure 10:**
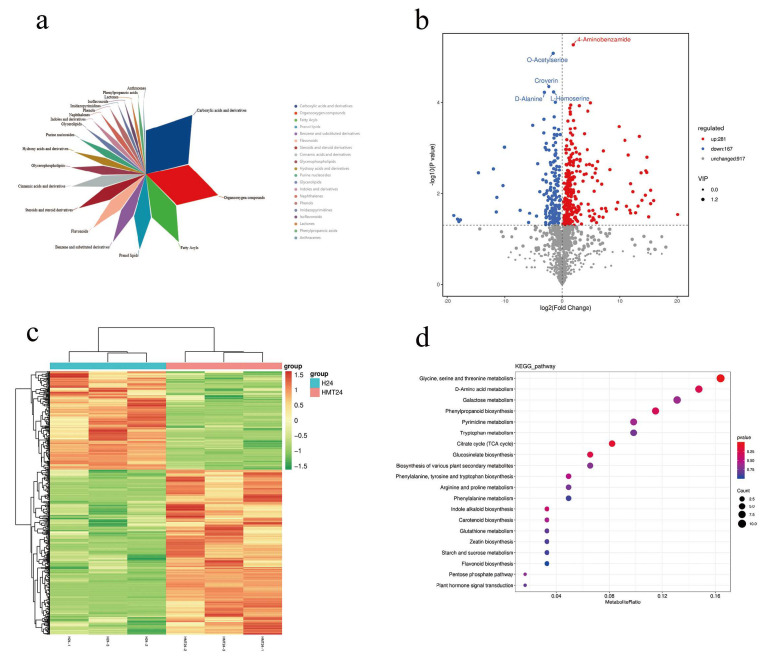
Screening, functional annotation, and enrichment analysis of DMs. (**a**) Volcano plot of DMs. (**b**) Classification petal plot of DMs. (**c**) Cluster analysis of DMs. (**d**) Enrichment analysis of DMs.

Subsequent metabolomic analysis identified a total of 448 differential metabolites at 24 h in both the HS and HMT treatments, with 281 upregulated and 167 downregulated. The differential metabolites were grouped into 20 categories, including “carboxylic acids and their derivatives”, “organic oxygen compounds”, “fatty acyls”, “prenol lipids”, “benzenes and their derivatives”, and “flavonoid compounds”. These findings suggest that heat stress induces a diverse accumulation of metabolites. Figure 10c illustrates that melatonin treatment induced significant differences between the two treatments in response to heat stress. KEGG pathway enrichment analysis of the differential metabolites revealed significant enrichment in several pathways, including “glycine, serine, and threonine metabolism”, “D-amino acid metabolism”, “galactose metabolism”, “phenylpropanoid biosynthesis”, and “tryptophan metabolism”.

### 2.11. Integrated Transcriptomic and Metabolomic Analysis

**Figure 11:**
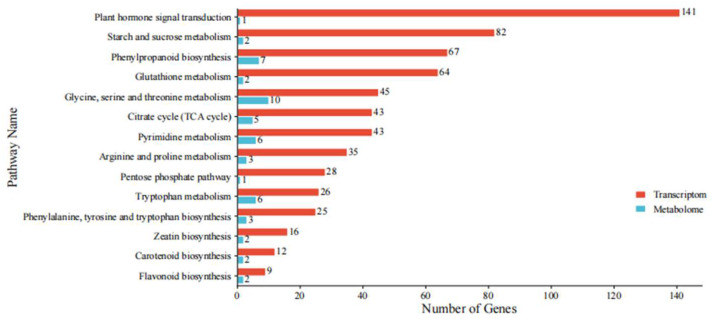
The common pathway diagram of the joint transcriptomic and metabolomic analysis.

To gain deeper insights into the molecular mechanisms underlying the promotion of maize seed germination by melatonin under heat stress, we undertook a comprehensive correlation analysis of the differential genes and metabolites between the HS and HMT treatment groups. Based on the KEGG enrichment results, we identified 14 metabolic pathways commonly enriched in both differential genes and metabolites, with a primary focus on pathways related to plant hormone signal transduction, starch and sucrose metabolism, and other key metabolic processes, illustrated in Figure 11.

#### 2.11.1. Analysis of Plant Hormone Synthesis and Signal Transduction Pathways

**Figure 12:**
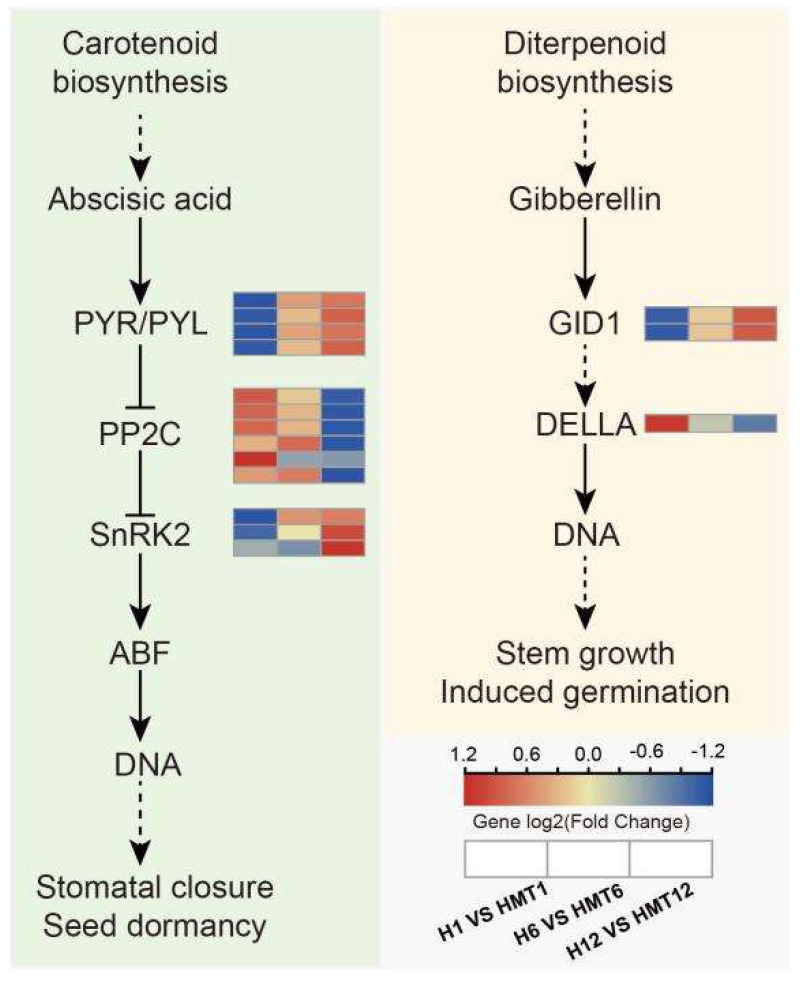
Analysis of plant hormone synthesis and signal transduction pathways in maize seeds under HS.

Based on early measurements of endogenous hormones and the enrichment analysis of DEGs, this study suggests that melatonin primarily influences seed germination through interactions with plant hormones. The transcriptomic KEGG pathway analysis revealed significant upregulation of “plant hormone signal transduction” (ko04075), with GA and ABA identified as the two most critical hormones in seed germination, as depicted in Figure 12. Relevant DEGs involved in these pathways were selected, and their expression patterns and regulatory roles were analyzed. Specifically, 13 DEGs were found to be involved in the ABA signaling pathway, with *PYR/PYL* genes predominantly upregulated. These genes showed notable fluctuations during the early germination stage, with the HMT treatment exhibiting higher levels than the HS treatment. In contrast, *PP2C* genes were mainly downregulated, limiting ABA’s influence on maize germination during this regulatory phase, thereby reducing its inhibitory effect and indirectly promoting seed germination. Notably, differences between the two treatments were observed, with *SnRK2* genes displaying an upregulation trend. In the GA signaling pathway, three DEGs were identified, including *GID1*, as a reliable regulatory factor that functions as a receptor that binds to GA and activates downstream signaling cascades. In this study, it was found to be upregulated in expression. *DELLA* proteins, which serve as crucial negative regulators of GA signaling, were notably downregulated in this study. This downregulation likely enhances GA signaling, thereby facilitating the gradual breaking of seed dormancy. Additionally, measurements of GA hormone levels, including *GA1*, *GA4*, *GA8*, and *GA19*, revealed an overall declining trend, with the HMT treatment exhibiting significantly lower levels than the HS treatment and a more rapid decrease. Overall, these findings suggest that melatonin enhances heat tolerance and promotes germination mainly by regulating the expression of genes associated with the GA and ABA signaling pathways.

#### 2.11.2. Analysis of Starch and Sucrose Metabolism Pathways

**Figure 13:**
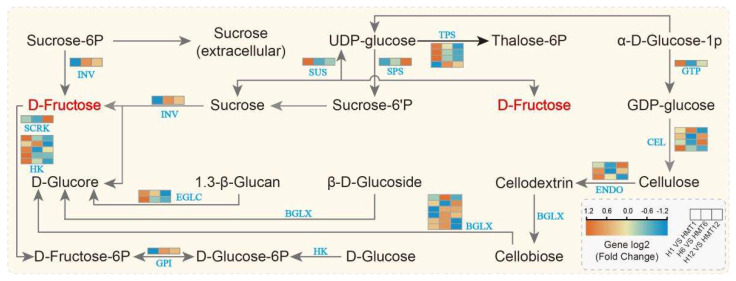
Starch and sucrose metabolism pathways in maize seeds under HS.

In the starch and sucrose metabolism pathway (ko00500), as shown in Figure 13, we found that melatonin co-regulated 37 DEGs and 2 differential metabolites. It was evident that genes involved in the synthesis of starch- and sucrose-degrading enzymes were predominantly downregulated throughout maize germination, while genes associated with glucose synthesis were mainly upregulated. For instance, genes encoding α-amylase and β-amylase (*AMY*) showed a slower decrease during the early germination stage, followed by a rapid decline later, with a similar pattern observed for the starch-converting gene Inv. Furthermore, melatonin upregulated genes associated with glycoside hydrolase (*ENDO*), glucose-1-phosphate adenylyltransferase (*GLGC*), hydroxyl ester lipase (*CEL*), β-glucosidase (*BGL*), and hexokinase (*HK*) in the EMP pathway, while it downregulated trehalose-6-phosphate synthase (*TPS*). Sucrose synthase (*SUS*) facilitates the conversion of sucrose into UDP-glucose and D-fructose in the presence of uridine diphosphate (*UDP*). Melatonin treatment resulted in a marked elevation of D-fructose levels. These results suggest that melatonin enhances the breakdown of starch and sucrose and promotes glucose synthesis under heat stress, providing the necessary energy to support seed germination.

#### 2.11.3. Analysis of the Phenylpropanoid Biosynthesis Pathway

**Figure 14:**
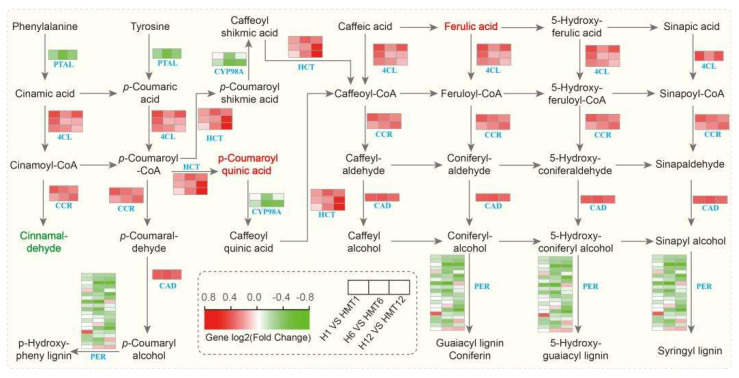
Analysis of the phenylpropanoid biosynthesis pathway in maize seeds under HS.

The phenylpropanoid biosynthesis pathway (ko00940) plays a crucial role in plants’ response to environmental stress by producing lignin monomers and phenolic metabolites that protect cellular structures. In the pathway of Figure 14, we identified 49 DEGs and 4 DMs. The enzyme phenylalanine/tyrosine ammonia-lyase (*PTAL*), which produces cinnamic acid, showed upregulated *PTAL* expression during this conversion. Additionally, 4-coumarate-CoA ligase (*4CL*), responsible for converting hydroxycinnamic acid into p-coumaroyl-CoA, showed significant upregulation, with the most notable increase observed in the later stages of germination. The synthesis of phenolic compounds in this pathway primarily involves hydroxycinnamoyl-CoA transferase (*HCT*) and cinnamoyl-CoA reductase (*CCR*), both of which demonstrated downregulation in expression, along with cinnamyl alcohol dehydrogenase (*CAD*). Conversely, the 3′-hydroxylase *CYP98A* displayed an increasing trend. While the metabolite cinnamaldehyde, produced during the later stages of *CCR* expression, generally decreased, it was elevated under melatonin treatment. Similarly, p-coumaroylquinic acid, generated from *HCT* expression, increased significantly, with even higher levels observed following melatonin application. Furthermore, 25 peroxidase genes were involved in the final steps of lignin synthesis, facilitating the formation of various lignin monomers, such as H-lignin. Melatonin treatment significantly enhanced the efficiency of lignin synthesis within maize seeds, thereby reinforcing cell wall defenses and exerting antioxidant effects under heat stress conditions. In summary, our results suggest that melatonin fortifies cell wall defenses, thereby enhancing resilience in heat stress environments.

## 3. Discussion

Heat stress significantly impairs crop growth, particularly during seed germination, posing a major challenge to maize productivity. During seed imbibition, metabolic processes are rapidly activated. These processes include increased respiration, enhanced enzymatic activity, accelerated starch hydrolysis, and rapid cell division, all of which facilitate radicle emergence and seed germination [37]. The germination process is influenced by external environmental factors and is tightly regulated by endogenous hormones. Over the years, melatonin has emerged as a crucial regulator of plant responses to abiotic stress [38,39,40,41]. Previous studies have shown that the effects of melatonin on cotton seed germination rates are dose-dependent [42]. This study demonstrates that treatment with 50 μM melatonin significantly enhances maize seed germination potential by promoting the elongation of both the radicle and the coleoptile. Additionally, melatonin regulates GA levels, which stimulates α-amylase synthesis and its hydrolytic activity on starch, thereby accelerating the germination process [43]. Under salt stress, melatonin has been reported to increase the contents of α-AMS and β-GAL in cotton seeds [44]. In this study, melatonin treatment significantly increased the activities of α-AMS, α-GAL, and β-GAL in maize seeds, facilitating starch degradation and sugar conversion to provide essential nutritional support for germination.

Studies have demonstrated that heat stress often results in excessive accumulation of ROS, causing damage to cell membranes and other cellular structures [45,46]. Melatonin notably stimulates the antioxidant enzyme system in maize seeds, boosting the activities of SOD, CAT, and POD, which helps to effectively eliminate excess ROS and reduce oxidative stress-related damage [47]. This antioxidant mechanism is considered a central pathway for plant resistance to environmental stresses [48]. Additionally, the accumulation of osmoprotectants is crucial in maintaining cellular osmotic balance and protecting membrane stability [49,50]. Melatonin can reduce oxidative stress through multiple pathways, primarily by scavenging ROS, including H_2_O_2_ and O_2_^−^ [51], thereby lowering MDA levels, which are a common indicator of oxidative damage under stress conditions [9]. The present study demonstrates that melatonin treatment significantly enhanced the activities of SOD, POD, and CAT in maize seeds, along with increasing the levels of soluble sugars and proline. Melatonin also reduced H_2_O_2_, O_2_^−^, and MDA levels, further supporting its role in enhancing antioxidant defenses and mitigating membrane damage under heat stress. Furthermore, qRT-PCR analysis confirmed that melatonin modulated the expression of genes associated with ROS scavenging and osmotic regulation, contributing to the enhanced heat tolerance of maize seeds. These results align with previous studies [26].

ABA and GA are essential hormones that regulate seed germination and stress responses, with their antagonistic roles being critical in controlling seed germination and dormancy [52]. Melatonin has been shown to regulate the balance between GA and ABA by stabilizing *DELLA* proteins [53,54]. In Arabidopsis, during the early stages of germination, the expression of ABA signaling pathway genes, including *ABI3*, *ABI4*, and *ABI5*, is significantly downregulated [55], which correlates with a reduction in endogenous ABA levels [56]. Melatonin treatment has been shown to upregulate the expression of *CYP707A1* and *CYP707A2* while enhancing the expression of *GA20ox* and *GA3*, thereby promoting seed germination [57]. Herein, melatonin treatment significantly reduced ABA levels and increased GA levels in maize seeds, consistent with previous findings [44]. These findings suggest that melatonin enhances heat tolerance in maize not only through its antioxidant properties but also by modulating the GA and ABA signaling pathways, as evidenced by the changes in gene expression in this study [58,59,60]. In the ABA signaling pathway, ABA receptors (*PYR/PYL/RCAR*) activate the expression of *SnRK2* by inhibiting the ABA-negative regulator *PP2C*, thereby regulating downstream ABA-responsive genes. In this study, qRT-PCR analysis revealed that melatonin significantly modulated the expression of several key genes, including *GA3*, *GA20ox*, *ZEP*, *NCED*, and *CYP707A*, in maize seeds. The transcriptomic analysis further revealed that *ZmPYR/PYL* genes were significantly upregulated in the HMT treatment during the early germination phase, while *ZmPP2C* expression showed a downregulation trend, effectively reducing ABA’s inhibitory effects on maize germination. Furthermore, *SnRK2* genes exhibited an upward trend in the HMT treatment.

Research has shown that starch and sucrose metabolism, along with α-linolenic acid metabolism, play a role in improving seed heat tolerance by regulating glycolysis pathways and hormone signaling networks [61,62]. During seed germination, the breakdown of stored substances such as starch and sucrose provides essential energy for growth [63]. Essential enzymes in starch hydrolysis, such as invertase (*Inv*), α-amylase, and β-amylase (*AMY*), are crucial for the sequential breakdown of starch into maltose. In quercus glauca thunb seeds, the activity and mRNA levels of *β-AMY* were significantly upregulated, whereas in Eucommia seeds, *Inv* and *AMY* were significantly upregulated. In this study, melatonin treatment significantly upregulated the expression of *Inv* and *AMY* in maize seeds. In addition, the regulation of energy metabolism is essential for seed germination. For instance, hexokinase (*HK*), a key enzyme in the EMP pathway, exhibits significantly elevated expression during the germination of rice and maize seeds [64,65]. This study found that melatonin treatment similarly upregulated *HK* expression, further highlighting its positive role in promoting energy conversion and supply. Moreover, trehalose, a key intermediate metabolite in starch and sucrose metabolism, is known to confer protective effects in plants under heat stress [66]. Our findings indicate that melatonin treatment significantly inhibited the expression of *TPS* in the trehalose synthesis pathway while promoting glucose accumulation, suggesting that melatonin optimizes carbohydrate storage and utilization, thereby promoting seed germination through the regulation of sugar metabolism pathways.

The phenylpropanoid biosynthesis pathway plays a critical role in plant secondary metabolism, contributing to plant growth, stress tolerance, and responses to environmental changes. This pathway is initiated by phenylalanine deaminase (*PTAL*) catalysis, which is positively influenced by melatonin. Previous studies have reported that the expression of the *PTA1* gene peaks during lignin synthesis, while the expression of *PAL* is significantly lower than that of *PTAL1* [67]. In this study, *PTAL* expression was significantly upregulated, while *PAL* expression remained unchanged, and the difference in their regulation under heat stress following melatonin treatment may be attributed to their distinct regulatory mechanisms. The *4CL* gene catalyzes the conversion of p-coumaroyl-CoA, which is subsequently transformed into methoxy and hydroxy derivatives. Oxidases, transferases, and reductases further catalyze these derivatives to produce flavonoids, anthocyanins, and other bioactive substances [68,69]. Herein, heat stress-induced upregulation of *4CL* genes enhanced lignin synthesis, thereby accelerating cell wall strengthening and boosting antioxidant defenses. These findings are consistent with previous research, which suggests that melatonin not only promotes lignin synthesis under heat stress but also regulates other key steps in the phenylpropanoid pathway, providing comprehensive support for maize seed germination.

## 4. Materials and Methods

### 4.1. Reagents

Melatonin (MT) was sourced from Yuanye Bio-Technology Co., Ltd., (Shanghai, China). All chemicals employed were of analytical grade.

### 4.2. Plant Material and Growth Conditions

The experiment was conducted at Hebei Agricultural University (Baoding, Hebei; 38.82° N, 115.45° E) using the heat-sensitive maize variety Xianyu 1466, with seeds harvested in 2023. Uniform, mature maize seeds were carefully chosen, rinsed thoroughly with distilled water to eliminate surface impurities, and disinfected by soaking in a 5% NaOCl solution for 15 min. Subsequently, the seeds were rinsed three times with distilled water and then immersed in melatonin solutions at concentrations of 25, 50, 75 and 100 μmol/L at 25 °C for 12 h, with distilled water serving as the control. Each treatment included 20 seeds and was replicated biologically with 6 independent samples. The treated seeds were evenly distributed in Petri dishes, each lined with two layers of sterilized filter paper.

The germination temperature was set to 42 °C, with 25 °C used for the control. The relative air humidity in the incubator was maintained at 60%. The high-temperature incubator was set to 42 °C/25 °C (day/night), while the control incubator maintained a constant 25 °C throughout the day. Seed germination was observed and documented daily at 8:00 a.m. Samples were taken at different germination stages, quickly frozen in liquid nitrogen, and stored at −80 °C for later physiological analysis. The experimental groups were as follows: CK (control treatment), MT (control melatonin treatment), HS (heat stress treatment), and HMT (heat stress melatonin treatment).

### 4.3. Assessment of Germination Rate, Potential, and Index

Germination was monitored at 12 h and on days 1, 2, 3, 4, 5 and 6. Seeds were considered germinated if their roots exceeded 2 mm in length. The calculations for the following indices followed [70]:

Germination rate (%) = (number of germinated seeds on day 5/total number of seeds) × 100.

Germination potential (%) = (number of germinated seeds on day 3/total seeds) × 100.

Germination index = ∑(Gi/Ti), where Gi is the germination rate on day i and Ti is the day number.

Seed vigor index = root length on day 5 × germination index.

### 4.4. Measurement of Root and Shoot Lengths in Embryos

On day 3, three germinated seeds from each group were randomly selected, and the root and shoot lengths of the embryos were measured using a vernier caliper [71].

### 4.5. Physiological, Biochemical, and Endogenous Hormone Quantification

The contents of α-AMS, α-GAL, β-GAL, proline, soluble sugars, MDA, H_2_O_2_, and O_2_^−^, as well as the O_2_^-^ rate and POD, SOD, and CAT activities, were measured using assay kits purchased from Suzhou Keming Biotechnology Co., Ltd., (Suzhou, China) The contents of abscisic acid (ABA) and gibberellins (GAs) were quantified using ELISA kits obtained from Shanghai Chaorui Biotechnology Co., Ltd., (Shanghai, China) Each treatment included three biological replicates. The indices listed above were determined using an enzyme marker Epoch 2 (Bio Tek Instruments, San Diego, CA, USA).

### 4.6. Transcriptome and Metabolome Analyses

Seed samples from the HS and HMT treatments were collected at 1, 6, and 12 h, with three replicates per treatment. Transcriptome sequencing was performed by AnnoBiotech (Beijing, China) Co., Ltd., utilizing the Illumina HiSeq 1000 System. RNA sequencing libraries were prepared following the manufacturer’s protocol. Data quality was evaluated using Fastp, and the clean data were aligned to the maize reference genome (https://www.maizegdb.org (accessed on 1 March 2024). Gene expression levels were determined by FPKM, and differential expression was assessed using DESeq2, with criteria of |log2FC| ≥ 1 and FDR < 0.05. KEGG enrichment analysis was subsequently conducted, applying a significance threshold of q-value < 0.05.

For the metabolome analysis, seed samples were collected at 24 h, with three replicates per treatment. Metabolite extraction was carried out using a UPLC-ESI-MS/MS system (UPLC, Waters Acquity I-Class PLUS; MS, Applied Biosystems QTRAP 6500+). Data analysis involved PCA and Spearman correlation analysis to evaluate the reproducibility of the samples. Metabolites were categorized, and pathways were identified using the KEGG, HMDB, and LipidMaps databases. Statistical significance was determined by fold change (FC), *t*-test (*p* < 0.05), and VIP values derived from OPLS-DA models. Differential metabolites were selected based on criteria of FC > 1, *p*-value < 0.05, and VIP > 1. KEGG pathway enrichment was assessed using hypergeometric distribution testing.

### 4.7. Quantitative Real-Time PCR (qRT-PCR) Analysis

The qRT-PCR reaction mix (10 μL) consisted of 5 μL of 2 × SYBR Green Mix, 0.5 μL each of forward and reverse primers, 1 μL of 6 × diluted cDNA template, and 3 μL of ultrapure water. The cycling protocol involved initial denaturation at 95 °C for 30 s, followed by 40 cycles of a three-step amplification, namely 95 °C for 5 s, 57 °C for 10 s, and 72 °C for 20 s, with a subsequent melt curve analysis. Relative gene expression was calculated using the 2^−ΔΔCt^ method, and statistical significance was determined by Student’s *t*-test (*p* < 0.05). The qRT-PCR data were aligned with the RNA-seq results, with three biological replicates per treatment. The primer sequences used for qRT-PCR are provided in Supplementary Material S1.4.8. Statistics and Analysis.

Data were produced using Microsoft Excel 2021, analyzed for significance using the DPS 7.05 package and plotted using Origin 2021 software.

## 5. Conclusions

This research shows that melatonin considerably boosts the germination of maize seeds when subjected to heat stress by modulating the activity of antioxidant enzymes, the accumulation of osmoprotectants, hormone metabolism, signal transduction, and various metabolic pathways. These synergistic effects contribute to a strong physiological protection mechanism, thus enhancing heat tolerance in maize seeds. The results hold significant implications for agricultural practices, especially in managing crops amidst climate change challenges where heat stress presents a considerable obstacle. Additionally, this study paves the way for future research to investigate the wider applications of melatonin in different crops and its combination with other stress management techniques to enhance overall crop resilience.

## Data Availability

Data is contained within the article and Supplementary Material.

## Supplementary Materials

The following supporting information can be downloaded at: https://www.mdpi.com/article/10.3390/ijms26041608/s1.
